# Smart Sensors for Healthcare and Medical Applications

**DOI:** 10.3390/s21020543

**Published:** 2021-01-14

**Authors:** Domenico Formica, Emiliano Schena

**Affiliations:** 1 Unit of Neurophysiology and Neuroengineering of Human Technology Interaction (NeXT), Università Campus Bio-Medico di Roma, 00128 Rome, Italy; 2 Laboratory of Measurements and Biomedical Instrumentation, Università Campus Bio-Medico di Roma, 00128 Rome, Italy; e.schena@unicampus.it

This special issue on “Smart Sensors for Healthcare and Medical Applications” focuses on new sensing technologies, measurement techniques, and their applications in medicine and healthcare. We proposed this topic, being aware of the pivotal role that smart sensors can play for the improvement of healthcare services in both acute and chronic conditions as well as for prevention towards a healthy life and active aging. In this editorial we shortly describe the potential of smart sensors in the aforementioned applications, before moving on providing a general overview of the 24 articles selected and published in this special issue.

Recent advances in mechatronics, Internet of Things, wearable devices, and the miniaturization of sensors and electronics have significantly increased the capabilities of smart sensors enlarging the range of their applications. Among others, healthcare and medical fields have been strongly influenced by the evolution of these technologies, which are providing substantial contributions in several applications in the field [1,2,3,4]. In fact, smart sensors are increasingly offering novel solutions to several relevant challenges in healthcare, such as early detection of pathologies, or minimally invasive management and prevention of high-burden diseases (e.g., cardiovascular diseases and cancer) [5]. Furthermore, the development of miniaturized and lightweight smart sensors-based systems may be a key player in enabling a more rapid growth of unobtrusive and unsupervised approaches to home-rehabilitation and continuous monitoring of patients’ status.

The 24 articles (i.e., 1 review paper, 22 articles, and 1 letter) selected in this special issue cover a variety of topics related to the design, validation, and application of smart sensors to healthcare. Indeed, the accepted papers report on different technologies exploited to develop smart sensors, several applications in diagnostic and/or therapeutic processes, new systems to assess the performance of smart sensors, wearable systems, and new approaches to analyze biomedical signals.

The only review paper published in this special issue focuses on the principles of work and technologies used to develop systems for estimating the radiation dose in medical applications [6]. In fact, the use of radiation is crucial in many radiotherapeutic or imaging procedures, but the absorbed dose could cause secondary malignancies [7]. Therefore, the accurate monitoring of the exposure to radiation is mandatory for both patients and clinicians. This work [6] allows the reader to have a global vision of the current landscape of photonic instruments used for dosimetry.

Five articles investigate new measuring systems and data analysis techniques for monitoring cardiac and/or respiratory activities, as well as syndromes related to their deficiencies [8,9,10,11,12]. In [8], the respiratory activity has been investigated as a parameter related to sleep disorders; the authors analyzed respiration and body movements by a non-contact technique, based on thermal imaging cameras, without disturbing human sleep. Their analysis was performed on 16 volunteers showing good performances in terms of respiratory rate estimation. In [9], a multi-sensor module to monitor the presence of breath by accelerometers, ECG, and SpO2 was proposed for monitoring patients affected by obstructive sleep apnea-hypopnea syndrome. The hardware solution was combined with novel neural network classification techniques for identifying obstructive sleep apnea. A further non-contact method for estimating the respiratory rate has been proposed in [10]. The system, consisting of a laptop’s built-in RGB camera and a custom algorithm, was assessed on 12 healthy volunteers by comparing the results with a gold standard. The agreement between the proposed method, and the reference one was promising. The importance of cardio-respiratory monitoring was also investigated in precision sports [11]. The system was proposed to monitor respiratory rate and heart rate on archers, since the breathing and heartbeat can influence the stability and, thus, athlete’s performance. A custom wearable system based on fiber optic sensors (i.e., fiber Bragg grating, FBG, sensors) was assessed on both 9 volunteers and in a real-life scenario on an archer during shooting session. The use of FBGs was also exploited for detecting pulse wave and estimating blood pressure in [12] and assessed on four healthy volunteers. The authors highlighted the importance of their solution to improve accuracy and repeatability in terms of pulse wave signal.

A second group of five papers propose methods to assess or predict patients’ conditions by monitoring different parameters (e.g., physiological parameters, physiological behaviors, and motion behaviors) [13,14,15,16,17]. The first article explores the use of optical sensors for pressure ulcer prevention [13]. Data collected by the system were used as feedback of an active pressure relief system by regulating the air flow. The authors demonstrated that the proposed system is able to reduce the pressure in a scenario mimicking the real condition. The second article focuses on a torsional wave sensor for estimating the cervical elasticity on pregnant women [14]. This novel technique was evaluated on 18 single-pregnant women showing that it is safe and allows quantifying cervical shear stiffness. Moreover, results proved that cervical stiffness can be considered a valuable predictor of gestational age at the moment of evaluation. The third article proposes a measurement system based on fiber optic sensors to detect grasping actions in newborns [15]. The authors focused on fiber optic technology since it allowed developing an MR compatible system. This feature is important because the use of the proposed system during functional magnetic resonance imaging (fMRI) helps to relate the brain activity to the grasping actions. The combination of these two analyses may provide important insights on how functional outcomes can be improved following cerebral injury. In [16], authors proposed a novel method based on artificial neural networks to detect epileptic seizures using data of 30 subjects from a public database. EEG, submentalis and bilateral anterior tibialis EMG, and an earlobe PPG were used showing promising results in terms of detection of epileptic seizures. A custom EEG-electrode holder infrastructure is presented in [17]. A low-end consumer 3D printer was used to manufacture all the elements (e.g., sensor-positioning ring, inter-ring bridge, and bridge shield), and the practicability of the assembled headset was validated on fifteen volunteers demonstrating the potential of a cost-efficient electrode-holder assembly infrastructure.

Seven articles focused on patients’ motor functions for potential application in rehabilitation or in the assessment of their functional impairment [18,19,20,21,22,23]. The first article proposes a novel contactless method, based on electrostatic field, for extracting gait features of hemiplegic patients [18], together with an improved version of the traditional detrended cross-correlation analysis to analyze the signal collected on 10 hemiparetic patients and 10 healthy volunteers. The authors found that the proposed method can quantify the gait difference between the hemiparetic patients and the healthy controls. The second paper proposes a solution to support medical personnel in the assessment of patients with spinal cord injury [19]. The authors present a system based on 3 RGB-D cameras (Microsoft Kinect v2) to assess patients’ movements using a wheelchair. The system allows analyzing the pushing cycle, and other key movements useful to evaluate the patient’s performance, thus, capable of supporting the clinician in the definition of the rehabilitation process. The system was assessed on patients for a total of 138 acquisitions showing promising results, also considering the low cost of the proposed system compared to the more expensive motion capture systems often used in similar applications. The third article finds its motivation in the development of a game-based system for upper-limb cerebral palsy [20]. The authors highlighted the importance of an accurate estimation of patient motor performance in order to have an effective game-based rehabilitation system. They used a Kinect sensor to monitor arm movements, focusing on the shoulder joint. The system was assessed on two groups of cerebral palsy and typically developing children showing good results in the movements evaluation of the right arm during the proposed rehabilitation sessions. The fourth article investigates a system for monitoring a medical exam (i.e., 30 s chair stand test) used to assess the functional status of elderly people [21]. The aim was to develop a system able to achieve the counts of sit-to-stand transitions without the supervision of a physician, and to guide the people through the whole exam via a home care application. The abovementioned count was performed by a simple wearable system based on an elastic band worn around the subject’s leg. The system was assessed on seven elderly subjects showing the capability of the system to identify all the sit-to-stand transitions. The fifth work proposes a wearable system for monitoring body acceleration and electromyography on patients affected by multiple sclerosis [22]. The system was tested on 40 patients and 15 healthy volunteers during the timed 10 min walking test under three different conditions: standard (i.e., wearing shoes), reduced grip (i.e., wearing socks), and increased cognitive load (i.e., backward-counting dual-task). The knowledge of both muscle activity and kinematics during walking may be beneficial to monitor the disease progression and the efficiency of rehabilitation for multiple sclerosis patients. In addition, results showed that walking tests wearing socks should be discouraged to prevent falls for these patients. The sixth article of this group focuses on the impact that the simultaneous knowledge of morphology and muscle activation can have in understanding the impairment status, thus providing a targeted rehabilitation treatment [23]. The study investigates a method that uses the morphological information contained in the MRI scan to build an electrical lumped model of the conductive volume to provide an estimation of all muscles’ activation. The last research paper of this group focuses on monitoring patients’ activity by a wearable system based on three-axis accelerometer and a triple-axis gyroscope [24]. Data collected on the patients are intended to be used to adjust the oxygen flow delivered by portable oxygen concentrator in patients with COPD and respiratory failure. The system was assessed on 18 patients showing a significant reduction of the number of desaturation events.

A solution for dietary monitoring in terms of food mass and intake (FMI) based on a ring-type sensor has been proposed in [25]. This evaluation is important since the knowledge of FMI can be important to assess the physical condition of a person. The proposed solutions allowed the wearable system to optimize its sensitivity. The authors highlighted the potential of this solution to support the prevention of obesity and metabolic syndrome.

The development of a system based on multiple sensors (i.e., a magnetic door sensor, a motion detection sensor, and two time of flight sensors) to estimate the duration of patient-physician contact (consultation time) is the focus of [26]. The knowledge of this parameter may help in the optimization of the treatment processes and on the healthcare service. The system was assessed in two scenarios with preliminary experiments to evaluate the quality of the estimated time.

A mechatronic platform for detection of cancer nodules has been proposed in [27]. The system uses multi-sensors data providing mechanical stiffness and Ultrasound impedance of the tissue under exam. The system was assessed using phantoms mimicking diseased tissues showing promising results (the system correctly identified the tissue in the 90.3% of the cases).

The study reported in [28] grounds its motivation in the reduction of measurements error of a type of force/pressure sensor (i.e., force sensitive resistors, FSR) largely used in applications related to the pressure map at the interface of a prosthetic socket and a residual limb. Authors reported a regression-based method to calibrate these sensors considering different surfaces of contact (i.e., flat or curved surfaces). Different algorithms were assessed to improve the accuracy of this type of sensor.

The relevant problem in clinical practice of errors in the placement of ECG electrodes, that can cause significant signal changes, has been faced in [29]. The study proposed a method to simulate all possible ECG reversals and applied the proposed novel algebraic transformation in the standard 12-lead ECG setup.

The unique research paper with a novel system to assess the performance of sensors is reported in [30]. The study explores the development of a novel movement simulator devoted to assessing the performance of wearable systems for monitoring fetal movements. The rationale behind this study is motivated by the clinical importance of fetal movements to assess the fetal health and the difficulty of assessing the performance of the proposed system in preclinical stage.

In summary, the special issue “Smart Sensors for Healthcare and Medical Applications” reports a wide collection of articles focused on smart sensors and their applications in healthcare. The large part of the contributions is expected to consolidate the use of smart sensors in medicine and healthcare in order to support physiological monitoring, to assist rehabilitation processes, to validate medical devices, and to optimize performance of measuring systems in a variety of fields.

## Data Availability

Not applicable.
